# Quality of life and related factors among chronic hepatitis B-infected patients: a multi-center study, Turkey

**DOI:** 10.1186/s12955-016-0557-9

**Published:** 2016-11-03

**Authors:** Zehra Karacaer, Banu Cakir, Hakan Erdem, Kenan Ugurlu, Gul Durmus, Nevin Koc Ince, Cinar Ozturk, Rodrigo Hasbun, Ayse Batirel, Esmeray Mutlu Yilmaz, Ilkay Bozkurt, Mustafa Sunbul, Aynur Aynioglu, Aynur Atilla, Ayse Erbay, Ayse Inci, Cigdem Kader, Elif Tukenmez Tigen, Gokhan Karaahmetoglu, Seher Ayten Coskuner, Ebru Dik, Huseyin Tarakci, Selma Tosun, Fatime Korkmaz, Servet Kolgelier, Fatma Yilmaz Karadag, Serpil Erol, Kamuran Turker, Ceyda Necan, Ahmet Melih Sahin, Pinar Ergen, Gulsen Iskender, Pinar Korkmaz, Esma Gulesen Eroglu, Yasemin Durdu, Mehmet Ulug, Suna Secil Deniz, Filiz Koc, Saygın Nayman Alpat, Nefise Oztoprak, Omer Evirgen, Hamdi Sozen, Mustafa Dogan, Selcuk Kaya, Safak Kaya, Mustafa Altindis, Emel Aslan, Recep Tekin, Busra Ergut Sezer, Kevser Ozdemir, Gulden Ersoz, Ahmet Sahin, Ilhami Celik, Emsal Aydin, Aliye Bastug, Rezan Harman, Hacer Deniz Ozkaya, Emine Parlak, Ilknur Yavuz, Suzan Sacar, Senol Comoglu, Ercan Yenilmez, Fatma Sirmatel, Ilker Inanc Balkan, Yesim Alpay, Mustafa Hatipoglu, Affan Denk, Gunes Senol, Mehmet Bitirgen, Mehmet Faruk Geyik, Rahmet Guner, Ayten Kadanali, Ahmet Karakas, Mustafa Namiduru, Hatice Udurgucu, Rukiye Pinar Boluktas, Ergenekon Karagoz, Necati Ormeci

**Affiliations:** 1 Department of Infectious Diseases and Clinical Microbiology, Etimesgut Military Hospital, Ankara, Turkey; 2 Institute of Public Health, Hacettepe University Faculty of Medicine, Ankara, Turkey; 3 Department of Infectious Diseases and Clinical Microbiology, Gulhane Military Medical Academy, Ankara, Turkey; 4 Department of Infectious Diseases and Clinical Microbiology, 25 Aralık State Hospital, Gaziantep, Turkey; 5 Department of Infectious Diseases and Clinical Microbiology, Sevket Yilmaz Training and Research Hospital, Bursa, Turkey; 6 Department of Infectious Diseases and Clinical Microbiology, Duzce University School of Medicine, Duzce, Turkey; 7 Department of Infectious Diseases and Clinical Microbiology, Recep Tayyip Erdogan University School of Medicine, Rize, Turkey; 8 Department of Infectious Diseases, The University of Texas Health Science Center at Houston, Medical School, Houston, TX USA; 9 Department of Infectious Diseases and Clinical Microbiology, Kartal Dr. Lutfi Kirdar Education and Research Hospital, Istanbul, Turkey; 10 Department of Infectious Diseases and Clinical Microbiology, Samsun Training and Research Hospital, Samsun, Turkey; 11 Department of Infectious Diseases and Clinical Microbiology, Ondokuz Mayis University School of Medicine, Samsun, Turkey; 12 Department of Infectious Diseases and Clinical Microbiology, Ataturk State Hospital, Zonguldak, Turkey; 13 Department of Infectious Diseases and Clinical Microbiology, Bozok University School of Medicine, Yozgat, Turkey; 14 Department of Infectious Diseases and Clinical Microbiology, Kanuni Sultan Suleyman Training and Research Hospital, Istanbul, Turkey; 15 Department of Infectious Diseases and Clinical Microbiology, Marmara University Pendik Training and Research Hospital, Istanbul, Turkey; 16 Department of Infectious Diseases and Clinical Microbiology, Gulhane Military Medical Academy Haydarpasa Training and Research Hospital, Istanbul, Turkey; 17 Department of Infectious Diseases and Clinical Microbiology, Bozyaka Training and Research Hospital, Izmir, Turkey; 18 Department of Infectious Diseases and Clinical Microbiology, Izmir Metropolitan Municipalities Esrefpasa Hospital, Izmir, Turkey; 19 Department of Infectious Diseases and Clinical Microbiology, Konya Training and Research Hospital, Konya, Turkey; 20 Department of Infectious Diseases and Clinical Microbiology, Yildirim Beyazit University Ankara Ataturk Training and Research Hospital, Ankara, Turkey; 21 Department of Infectious Diseases and Clinical Microbiology, Medeniyet University, Goztepe Training and Research Hospital, Istanbul, Turkey; 22 Department of Infectious Diseases and Clinical Microbiology, Haydarpasa Numune Training and Research Hospital, Istanbul, Turkey; 23 Department of Infectious Diseases and Clinical Microbiology, Bagcilar Training and Research Hospital, Istanbul, Turkey; 24 Department of Infectious Diseases and Clinical Microbiology, Pamukkale University School of Medicine, Denizli, Turkey; 25 Department of Infectious Diseases and Clinical Microbiology, Giresun State Hospital, Giresun, Turkey; 26 Department of Infectious Diseases and Clinical Microbiology, Ankara Oncology Training and Research Hospital, Ankara, Turkey; 27 Department of Infectious Diseases and Clinical Microbiology, Dumlupinar University Evliya Celebi Training and Research Hospital, Kutahya, Turkey; 28 Department of Infectious Diseases and Clinical Microbiology, Necmettin Erbakan University, Meram School of Medicine, Konya, Turkey; 29 Department of Infectious Diseases and Clinical Microbiology, Eyup State Hospital, Istanbul, Turkey; 30 Department of Infectious Diseases and Clinical Microbiology, Umit Hospital, Eskisehir, Turkey; 31 Department of Infectious Diseases and Clinical Microbiology, Kecioren Training and Research Hospital, Ankara, Turkey; 32 Department of Infectious Diseases and Clinical Microbiology, Osmangazi University School of Medicine, Eskisehir, Turkey; 33 Department of Infectious Diseases and Clinical Microbiology, Antalya Training and Research Hospital, Antalya, Turkey; 34 Department of Infectious Diseases and Clinical Microbiology, Mustafa Kemal University School of Medicine, Hatay, Turkey; 35 Department of Infectious Diseases and Clinical Microbiology, Sitki Kocman University School of Medicine, Mugla, Turkey; 36 Department of Infectious Diseases and Clinical Microbiology, Corlu State Hospital, Tekirdag, Turkey; 37 Department of Infectious Diseases and Clinical Microbiology, Karadeniz Technical University School of Medicine, Trabzon, Turkey; 38 Department of Infectious Diseases and Clinical Microbiology, Gazi Yasargil Training and Research Hospital, Diyarbakir, Turkey; 39 Department of Clinical Microbiology, Sakarya University School of Medicine, Sakarya, Turkey; 40 Department of Infectious Diseases and Clinical Microbiology, Dicle University School of Medicine, Diyarbakir, Turkey; 41 Department of Infectious Diseases and Clinical Microbiology, Denizli State Hospital, Denizli, Turkey; 42 Department of Infectious Diseases and Clinical Microbiology, Mersin University School of Medicine, Mersin, Turkey; 43 Department of Infectious Diseases and Clinical Microbiology, Gaziantep University School of Medicine, Gaziantep, Turkey; 44 Department of Infectious Diseases and Clinical Microbiology, Kayseri Training and Research Hospital, Kayseri, Turkey; 45 Department of Infectious Diseases and Clinical Microbiology, Kafkas University School of Medicine, Kars, Turkey; 46 Department of Infectious Diseases and Clinical Microbiology, Ankara Numune Training and Research Hospital, Ankara, Turkey; 47 Department of Infectious Diseases and Clinical Microbiology, Sani Konukoglu Hospital, Gaziantep, Turkey; 48 Department of Infectious Diseases and Clinical Microbiology, Karsıkaya State Hospital, Izmir, Turkey; 49 Department of Infectious Diseases and Clinical Microbiology, Ataturk University School of Medicine, Erzurum, Turkey; 50 Department of Infectious Diseases and Clinical Microbiology, Trabzon Kanuni Training and Research Hospital, Trabzon, Turkey; 51 Department of Infectious Diseases and Clinical Microbiology, Onsekiz Mart University School of Medicine, Canakkale, Turkey; 52 Department of Infectious Diseases and Clinical Microbiology, Umraniye Training and Research Hospital, Istanbul, Turkey; 53 Department of Infectious Diseases and Clinical Microbiology, Kasimpasa Military Hospital, Istanbul, Turkey; 54 Department of Infectious Diseases and Clinical Microbiology, Abant Izzet Baysal University School of Medicine, Bolu, Turkey; 55 Department of Infectious Diseases and Clinical Microbiology, Istanbul University Cerrahpasa School of Medicine, Istanbul, Turkey; 56 Department of Infectious Diseases and Clinical Microbiology, Balikesir University School of Medicine, Balikesir, Turkey; 57 Department of Infectious Diseases and Clinical Microbiology, Canakkale Military Hospital, Canakkale, Turkey; 58 Department of Infectious Diseases and Clinical Microbiology, Firat University School of Medicine, Elazig, Turkey; 59 Department of Infectious Diseases and Clinical Microbiology, Izmir Dr. Suat Seren Pulmonology and Pulmonary Surgery Training and Research Hospital, Izmir, Turkey; 60 Faculty of Health Sciences, Department of Nursing, Selahaddin Eyyubi University, Diyarbakir, Turkey; 61 Department of Infectious Diseases and Clinical Microbiology, Van Military Hospital, Van, Turkey; 62 Department of Gastroenterology, Ankara University School of Medicine, Ankara, Turkey

**Keywords:** Chronic hepatitis B infection, Quality of life, The Hepatitis B Quality of Life Instrument, Turkey

## Abstract

**Background:**

The aim of this study was to assess health-related quality of life (HRQOL) among chronic hepatitis B (CHB) patients in Turkey and to study related factors.

**Methods:**

This multicenter study was carried out between January 01 and April 15, 2015 in Turkey in 57 centers. Adults were enrolled and studied in three groups. Group 1: Inactive HBsAg carriers, Group 2: CHB patients receiving antiviral therapy, Group 3: CHB patients who were neither receiving antiviral therapy nor were inactive HBsAg carriers. Study data was collected by face-to-face interviews using a standardized questionnaire, Short Form-36 (SF-36) and Hepatitis B Quality of Life (HBQOL). Values equivalent to *p* < 0.05 in analyses were accepted as statistically significant.

**Results:**

Four thousand two hundred fifty-seven patients with CHB were included in the study. Two thousand five hundred fifty-nine (60.1 %) of the patients were males. Groups 1, 2 and 3, consisted of 1529 (35.9 %), 1721 (40.4 %) and 1007 (23.7 %) patients, respectively. The highest value of HRQOL was found in inactive HBsAg carriers. We found that total HBQOL score increased when antiviral treatment was used. However, HRQOL of CHB patients varied according to their socio-demographic properties. Regarding total HBQOL score, a higher significant level of HRQOL was determined in inactive HBV patients when matched controls with the associated factors were provided.

**Conclusions:**

The HRQOL score of CHB patients was higher than expected and it can be worsen when the disease becomes active. Use of an antiviral therapy can contribute to increasing HRQOL of patients.

## Background

There are approximately 250 million individuals with chronic hepatitis B (CHB) around the world [1]. Turkey is ranked as a lower intermediate-endemic country with a prevalence of hepatitis B virus (HBV) that varies between 3 and 10 % in different regions [1, 2]. As the prevalence of CHB increase, prevention by education and vaccination in those uninfected and maintaining a “good quality of life” in those infected is crucial.

Quality of life means the general perception of positive and negative aspects of an individual [3]. Health is a very substantial part of the general quality of life. The concept of health related quality of life (HRQOL) and its markers began to emerge in the 1980s. In the last few decades, in parallel to the recognized significance of the quality of life of patients, there has been an increase in the assessment of HRQOL among patients with chronic liver disease. Particularly in some countries, monitoring HRQOL in chronic hepatitis C patients has become a standard procedure [4]. Similarly, the awareness of the importance of HRQOL assessment in CHB has increased [5–12]. Unfortunately, based on the results obtained from a healthy population, some results point to a decrease in quality of life in CHB patients [5]. HRQOL may worsen due to the progression of viral hepatitis [7].

For many years, CHB patients were assessed for HRQOL using generic scales that were developed to measure the quality of life in the general population [5–11]. The development of the Hepatitis B Quality of Life Instrument (HBQOL) by Spiegel et al. [13] filled an important gap regarding the assessment of CHB patients, using a special scale for the disease. After this development, a small number of studies carried out with the HBQOL were reported in the literature [14, 15]. The quality of life in Turkey was found to be quite low in various studies where different scales were used in CHB patients [5, 11]. Unfortunately, there have been no studies of the Turkish population that assessed quality of life in CHB patients using the HBQOL and in a nationwide extent. Therefore, we planned to assess HRQOL of a wide and heterogeneous group of CHB patients using the HBQOL scale and considering different properties.

## Methods

### Study design

**Fig. 1:**
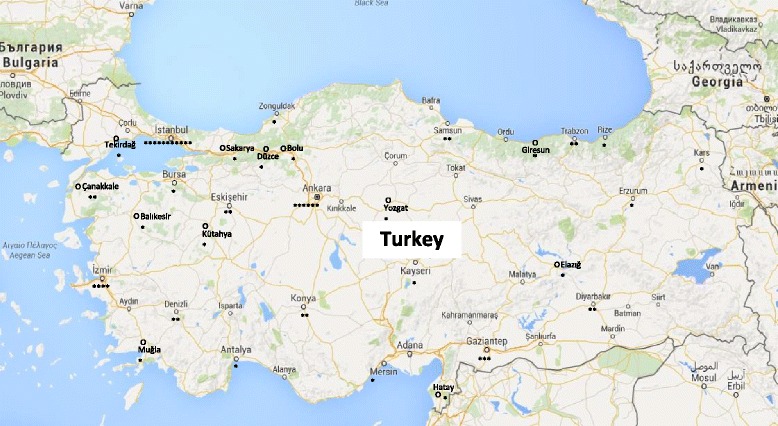
The cities where the participant centers in this study are located in Turkey. * The signs indicate the number of centers

This multi-center study was carried out prospectively between January 1 and April 15 2015, in 30 provinces in Turkey. A total of 57 centers comprised from 23 university hospitals, 21 training hospitals and 13 state hospitals participated in this study. Participating hospitals were widely scattered throughout the country, as presented in Fig. 1.

All CHB patients attending infectious disease outpatient clinics of participating hospitals over the study period were evaluated with a standardized interview. The study population included all who were eligible for participation, aged 18 years or over, and who provided informed consent. Accordingly, inactive carriers of HBsAg, chronic hepatitis B-infected patients, pre-cirrhotic cases (patients with fibrosis score five and without cirrhotic symptoms) and those being treated for chronic HBV infection were included in the study and were divided into the following three groups below:

- Group 1: HBsAg positive, HBeAg negative, with normal alanine aminotransferase level (ALT) levels, patients with a HBV Deoxyribonucleic acid (DNA) level < 2000 IU/ml and who were not receiving therapy.
- Group 2: Chronic hepatitis B patients from any of the groups receiving antiviral therapy.
- Group 3: Chronic hepatitis B patients who were not receiving antiviral therapy yet and who were not inactive HBsAg carriers.

ALT levels in our study were interpreted according to reference values used in the labs of each participating center.

The exclusion criteria were as follows: 1. Hepatitis C virus (HCV) infection, 2. Any type of cirrhosis, 3. Liver failure, 4. Liver cancer, 5. Presence of other liver disorders.

### Data collection tools

Data were collected using a standardized questionnaire (SQ) via a face-to-face interview, a Turkish version of Short Form-36 (SF-36) and a Turkish version of HBQOL. The study questionnaire included a total of 16 questions on sociodemographic characteristics and chronic HBV-related issues [14].

### Hepatitis B Quality of Life Instrument version 1.0-HBQOL

We used the hepatitis B quality of life instrument questionnaire produced by Spiegel in 2007 [13]. This questionnaire was subsequently verified by Pinar et al. [14] in a Turkish population. The subscales are listed as Psychological Well-Being, Anticipation Anxiety, Vitality, Stigma, Transmissibility and Vulnerability. There are 31 expressions having Likert type scoring from 1 to 5. The higher the scores in the subscales and in the total scale were interpreted as a better quality of life [13, 14].

The scale has undergone reliability and validity analyses, however, the Transmissibility subscale was found not to be reliable or valid in a Turkish population. It was assumed that after this subscale was removed the scale would become available and compatible for Turkish CHB patients [14].

At initiation of this study, researchers investigated the internal consistency of HBQOL subscales in the study population and showed high scores for each subscale. Cronbach’s alpha values were above 0.77 for subscales of Psychological Well-Being (0.93), Anticipation Anxiety (0.93), Vitality (0.84), Stigma (0.89) and Vulnerability (0.77) but were below acceptability (0.45) for Transmissibility subscales, confirming Pinar et al.’s earlier work [14]. Accordingly, transmissibility scores were not considered in calculation of the total score in this study, where total possible scores within the range of 28–152 were transferred to corresponding scores out of 100.

### Medical outcomes scale 36 item short form health survey-SF 36

This scale was developed by Ware and Sherburne in 1989 and was adapted into Turkish by Pinar et al. [16] and Kocyigit et al. [17]. The scale consists of 36 expressions. It contains two main headings, recognized as the Physical Component Summary and the Mental Component Summary. General Health, Vitality, Physical Functioning, Role Physical, Bodily Pain, Mental Health, Role Emotional and Social Functioning compose the subscales of the scale. Median scoring for each subscale is calculated by the Likert method, according to a standard SF-36 scoring algorithm [18]. Higher scores demonstrate better functionality or a much better life quality [16]. This survey was reprinted with permission from the RAND Corporation [18].

### Ethics

Declaration of Helsinki and Good Clinical Practice Guidelines were respected during the entire process of enrolling patients in the study and collecting/analyzing/reporting data. An approval from the local ethics committee was obtained for the study. Volunteering was the main option for participating in the study.

### Statistical analysis

All statistical analyses were conducted using the SPSS v.22.0 statistical software package (SPSS Inc., Chicago, IL, USA). Distributions of categorical variables were tested using Chi-square test. Mann-Whitney U and Kruskal-Wallis Tests were used for comparison of groups; Bonferroni corrections were calculated for binary comparisons. Normality assumption was tested for all continuous variables using Kolmogorov-Smirnov test: median, 25th and 75th percentile values are presented for continuous variables, if the normality assumption was not valid. Spearman Correlation Test was used for correlation between continuous variables.

Quality of life among patients with chronic HBV infection was further modeled using Covariance Analysis, where the total score from the HBQOL scale and activity of the disease were considered as the main dependent and independent variables, respectively, while gender, marital status, comorbidity, presence of HBeAg, serum ALT, current treatment status and type of hospital were treated as potential confounders. Statistical significance was set at *p* < 0.05, if not stated otherwise. Type I error was set at 0.0167 for bivariate comparisons where three or more groups were compared simultaneously.

## Results

### Sociodemographic characteristics of patients

Sociodemographic characteristics of patients and properties related to the disease are presented in Table 1. Significant differences were determined for all properties among patient groups (*p* < 0.05 for all).

**Table 1:** Distribution of socio-demographic and disease-related characteristics of patients with hepatitis B by disease status

Variable		Group-1 (*n* = 1529)	Group-2 (*n* = 1721)	Group-3 (*n* = 1007)	*p* value*	All patients (*n* = 4257)
Gender^a^	Male	805 (52,6)	1095 (63,6)	659 (65,4)	<0,001	2559 (60,1)
Age (years) ^b^		42 (32;52)	42 (32;52)	34 (25; 44)	<0,001	40 (30;50)
Marriage status^a^	Married	1256 (82,1)	1359 (79)	664 (65,9)	<0,001	3279 (77)
Education level^a^	Elementary school graduate and under	654 (42,8)	672 (39)	359 (35,7)	0,002	1685 (39,6)
	Middle school graduate	239 (15,6)	314 (18,2)	186 (18,5)		739 (17,4)
	High school graduate	366 (23,9)	376 (21,8)	250 (24,8)		992 (23,3)
	University graduate or higher	270 (17,7)	359 (20,9)	212 (21,1)		841 (19,8)
Regular income^a^	Present	893 (58,4)	1149 (66,8)	568 (56,4)	<0,001	2610 (61,3)
Any family member(s) with hepatitis B^a^	Present	740 (48,4)	983 (57,1)	606 (60,2)	<0,001	2329 (54,7)
Other chronic disease^a^	Present	373 (24,4)	484 (28,1)	196 (19,5)	<0,001	1053 (24,7)
Hospital type^a^	Training hospital	725 (47,4)	752 (43,7)	425 (42,2)	<0,001	1902 (44,7)
	University hospital	491 (32,1)	655 (38,1)	291 (28,9)		1437 (33,8)
	Other	313 (20,5)	314 (18,2)	291 (28,9)		918 (21,6)
Duration of diagnosis (months)^b^		84 (36;144)	84 (48;156)	72 (24;132)	<0,001	84 (36;144)
Period of therapy (months)^b^		N/A	36 (1–312, 12;60)	N/A		36 (1–312, 12;60)
ALT (U/L)^b^		21 (16;27)	26 (19;36)	34 (22;61)	<0,001	25 (18;36)
HBV DNA (IU/ml)^b^		80 (0;462,5)	0 (0;117)	7600 (2001;13 × 10^4^)	<0,001	76 (0;1700)
HBeAg status^a^	Positive	0	358 (20,8)	242 (24)	0,049	600 (14,1)

*p-value for comparison of distribution across three disease groups. Other chronic disease: respectively; hypertension, diabetes mellitus, psychiatric disorders, malignancy, chronic renal failure, congestive heart failure and others

^a^Number (percent), ^b^Median (% 25; % 75); *ALT* alanine aminotransferase, *HBV* Hepatitis B Virus, *DNA* Deoxyribonucleic acid

### SF-36 results

#### SF-36 results according to patient properties

All studied factors were found to be statistically significantly associated with Mental Component summary scores (*p* <0.05 for all). Similarly, all factors with the exception of marital status (*p* = 0.725) and HBeAg status (*p* = 0.082) were significantly associated with the Physical Component Summary score. Differences according to sociodemographic properties of patients with a subscale of the SF-36 scale are summarized in Table 2.

**Table 2:** Distribution of Short Form-36 subscale scores by some socio-demographic and disease-related characteristics of patients^a^

Variable	Physical functioning	Role physical	Role emotional	Vitality	Mental health	Social functioning	Bodily pain	General health	Physical component score	Mental component score
Gender	***p*** **:<0,001**	***p*** **:<0,001**	***p*** **:<0,001**	***p*** **:<0,001**	***p*** **:<0,001**	***p*** **:<0,001**	***p*** **:<0,001**	***p*** **:<0,001**	***p*** **:<0,001**	***p*** **:<0,001**
Male	90 (70;100)	100 (50;100)	100 (33,3;100)	55 (45;75)	72 (56;84)	87,5 (62,5;100)	100 (77,5;100)	65 (50;75)	79 (61,5;86)	74 (54,9;82,3)
Female	80 (55;100)	100 (25;100)	100 (0;100)	50 (35;65)	64 (48;80)	87,5 (50;100)	80 (55;100)	60 (40;70)	68,5 (47;84)	64,1 (44,6;80,8)
Marriage status	*p*:0,376	*p*:0,069	***p*** **:0,005**	*p*:0,292	***p*** **:<0,001**	***p*** **:<0,001**	*p*:0,378	*p*:0,073	*p*:0,725	***p*** **:0,009**
Married	90 (65;100)	100 (50;100)	100 (33,3;100)	50 (40;70)	68 (52;84)	87,5 (62,5;100)	90 (67,5;100)	65 (45;75)	76 (55,5;84)	71 (51,5;80,8)
Single	90 (65;100)	100 (43,7;100)	100 (33,3;100)	55 (40;75)	66 (48;80)	87,5 (50;100)	90 (67,5;100)	60 (40;75)	75 (54,5;86)	67,9 (46,9;81,2)
Education level	***p*** **:<0,001**	***p*** **:<0,001**	***p*** **:0,011**	***p*** **:<0,001**	***p*** **:<0,001**	***p*** **:0,013**	***p*** **:<0,001**	***p*** **:<0,001**	***p*** **:<0,001**	***p*** **:<0,001**
Elementery school and under	85 (55;100)	100 (25;100)	100 (33,3;100)	50 (40;65)	68 (52;84)	87,5 (62,5;100)	90 (65;100)	60 (45;70)	71,5 (52;84)	68,5 (48,8;80,8)
Middle school	85 (60;100)	100 (25;100)	100 (33,3;100)	55 (40;70)	68 (52;80)	75 (62,5;100)	90 (67,5;100)	60 (40;70)	72 (52;84)	68,4 (48,8;80,8)
High school	90 (70;100)	100 (50;100)	100 (33,3;100)	55 (40;75)	68 (52;80)	87,5 (62,5;100)	100 (67,5;100)	60 (45;75)	78 (56;86)	72,3 (50,5;81,9)
University	95 (80;100)	100 (75;100)	100 (33,3;100)	60 (45;75)	72 (56;84)	87,5 (62,5;100)	100 (77,5;100)	65 (50;75)	80 (66;87)	74,6 (56,6;83,3)
Regular income	***p*** **:<0,001**	***p*** **:<0,001**	***p*** **:<0,001**	***p*** **:<0,001**	***p*** **:<0,001**	***p*** **:<0,001**	***p*** **:<0,001**	***p*** **:<0,001**	***p*** **:<0,001**	***p*** **:<0,001**
Yes	95 (70;100)	100 (50;100)	100 (66,7;100)	55 (45;75)	72 (56;84)	87,5 (62,5;100)	100 (77,5;100)	65 (50;75)	80 (62;86)	74,8 (56,8;82)
No	80 (50;95)	75 (25;100)	66,7 (0;100)	50 (35;65)	64 (48;80)	75 (50;100)	77,5 (55;100)	55 (40;70)	67,5 (45;83)	62 (43,1;78,6)
Family members with hepatitis B	***p*** **:0,003**	***p*** **:0,003**	***p*** **:<0,001**	***p*** **:<0,001**	***p*** **:<0,001**	***p*** **:<0,001**	***p*** **:<0,001**	***p*** **:<0,001**	***p*** **:<0,001**	***p*** **:<0,001**
Present	85 (60;100)	100 (25;100)	100 (33,3;100)	50 (40;70)	68 (52;80)	87,5 (62,5;100)	90 (67,5;100)	60 (45,70)	73,5 (53,5;84)	68,3 (48,6;80,8)
Not	90 (70;100)	100 (50;100)	100 (33,3;100)	55 (40;70)	70 (52;84)	87,5 (62,5;100)	100 (67,5;100)	65 (45;75)	78 (57;86)	72,6 (52,8;82,1)
Received treatment for hepatitis B	***p*** **:<0,001**	***p*** **:0,001**	***p*** **:0,016**	*p*:0,164	*p*:0,481	*p*:0,763	*p*:0,712	***p*** **:0,006**	***p*** **:<0,001**	***p*** **:0,012**
Yes	85 (60;100)	100 (25;100)	100 (33,3;100)	55 (40;70)	68 (52;80)	87,5 (62,5;100)	90 (67,5;100)	60 (45;70)	74 (54,5;84)	69,4 (50;80,8)
No	90 (65;100)	100 (50;100)	100 (33,3;100)	50 (40;70)	68 (52;84)	87,5 (62,5;100)	90 (67,5;100)	65 (45;75)	77 56,5;85,8)	71,5 (50,5;81,4)
Other chronic disease	***p*** **:<0,001**	***p*** **:<0,001**	***p*** **:<0,001**	***p*** **:<0,001**	***p*** **:<0,001**	***p*** **:<0,001**	***p*** **:<0,001**	***p*** **:<0,001**	***p*** **:<0,001**	***p*** **:<0,001**
Present	75 (50;95)	75 (0;100)	66,7 (0;100)	50 (35;60)	64 (48;80)	75 (50;100)	77,5 (55;100)	55 (35;70)	64,5 (43;82)	63,3 (41,2;79,2)
Not	90 (70;100)	100 (50;100)	100 (33,3;100)	55 (45;75)	68 (52;84)	87,5 (62,5;100)	100 (77,5;100)	65 (50;75)	78 (60;86)	72,8 (53,6;82)
Hospital	***p*** **:<0,001**	***p*** **:<0,001**	***p*** **:<0,001**	*p*:0,291	***p*** **:<0,001**	***p*** **:<0,001**	***p*** **:<0,001**	***p*** **:<0,001**	***p*** **:<0,001**	***p*** **:<0,001**
Training hospital	95 (73,7;100)	100 (75;100)	100 (66,7;100)	50 (45;70)	72 (56;84)	100 (62,5;100)	100 (77,5;100)	65 (50;75)	80,5 (62,5;85)	75,7 (57,6;81,2)
University hospital	85 (60;100)	100 (25;100)	100 (33,3;100)	55 (40;70)	64 (52;80)	87,5 (62,5;100)	90 (67,5;100)	60 (45;75)	73 (52,7;84)	67,6 (48,8;80)
Other	80 (55;100)	75 (25;100)	66,7 (0;100)	55 (35;70)	64 (48;80)	75 (50;100)	77,5 (55;100)	60 (40;70)	67 (47,4;83)	61,2 (43,1;80)
HBeAg status	*p*:0,877	*p*:0,079	***p*** **:0,034**	*p*:0,135	*p*:0,357	***p*** **:0,014**	*p*:0,466	***p*** **:0,014**	*p*:0,082	***p*** **:0,010**
Positive	90 (65;100)	100 (25;100)	100 (33,3;100)	50 (40;70)	68 (52;80)	87,5 (50;100)	90 (67,5;100)	60 (45;70)	74 (54,5;84)	68,6 (48,7;80,8)
Negative	90 (65;100)	100 (50;100)	100 (33,3;100)	55 (40;70)	68 (52;84)	87,5 (62,5;100)	90 (67,5;100)	60 (45;75)	76 (55,5;85)	70,8 (50,8; 81)

^a^Values were not normally distributed; thus; median (% 25; % 75) scores are presented in cells, bold data reflected a statistical significance

A negative correlation was observed between the subscale scores of patient age and Physical Functioning and a positive correlation between the subscale scores of patient age and Role Emotional, Mental Health and Social Functioning. We also observed that the ALT level did not affect the subscale of Vitality, but elevated ALT did decrease the scores in the other subscales. It was also determined that Vitality, Physical Component Summary and Mental Component Summary scores were not affected by the HBV DNA level, but negatively affected the scores in other subscales. We found that the duration of diagnosis of the disease and the period of therapy did not affect SF-36 scores at all (Table 3).

**Table 3:** Correlation of Short Form-36 and Hepatitis B Quality of Life scores with selected patient and disease characteristics (r/p)^a^

Subscale	Age (years)	Duration of diagnosis (months)	Period of therapy (months)	ALT (U(L)	HBV DNA (IU(ml)
SF-36					
Physical functioning	−0,087/<0,001	0,009/0,553	−0,019/0,210	−0,037/0,015	−0,038/0,014
Role physical,	−0,018/0,235	0,002/0,906	0,013/0,382	−0,045/0,003	−0,035/0,024
Role emotional	0,052/0,001	0,002/0,895	0,016/0,303	−0,042/0,006	−0,043/0,005
Vitality	0,01/0,521	−0,034/0,025	0,013/0,399	−0,020/0,187	−0,028/0,068
Mental health	0,107/<0,001	0,004/0,781	0,024/0,123	−0,042/0,006	−0,051/0,001
Social functioning	0,062/<0,001	−0,019/0,216	0,027/0,076	−0,076/<0,001	−0,042/0,006
Bodily pain	−0,008/0,580	−0,003/0,844	0,035/0,023	−0,068/<0,001	−0,055/<0,001
General health	0,029/0,061	0,002/0,873	0,012/0,421	−0,044/0,004	−0,052/0,001
Physical component score	0,013/0,401	0,009/0,557	0,013/0,384	−0,044/0,004	−0,022/0,153
Mental component score	0,013/0,393	0,004/0,802	0,023/0,136	−0,032/0,038	−0,021/0,181
HBQOL					
Psychological Well-Being	0,010/0,526	0,004/0,771	0,020/0,195	−0,051/0,001	−0,012/0,421
Anticipation anxiety	0,106/<0,001	−0,003/0,827	−0,011/0,471	−0,043/0,005	−0,049/0,001
Vitality	0,063/<0,001	0,009/0,578	−0,010/0,514	−0,045/0,003	0,608/<0,001
Stigma	0,124/<0,001	0,050/0,001	0,003/0,843	−0,049/0,001	−0,052/0,001
Vulnerability	0,091/<0,001	0,004/0,789	−0,011/0,488	−0,048/0,002	−0,046/0,003
Total score	0,009/0,568	0,003/0,870	0,015/0,343	−0,049/0,001	−0,024/0,125

^a^Correlation coefficient (r) and relevant *p* value (in paranteses) are presented, *ALT* alanine aminotransferase level, *SF-36* Short Form-36, *HBQOL* Hepatitis B Quality of Life, *HBV* Hepatitis B Virus, *DNA* Deoxyribonucleic acid

#### SF-36 results according to patient groups

We found that Group 1 patients had the highest scores among the patient groups. We determined that there was no difference between Group 1 and Group 2 patients for subscales of Vitality and Bodily Pain (respectively, *p* = 0.060, *p* = 0.185) while a significant difference was found in other subscales. However, scores were similar only in Vitality subscales between Group 1 and Group 3 patients (*p* = 0.167), while a significant difference was found in others. There was a significant difference between Group 2 and Group 3 patients for subscales of Bodily Pain, Mental Health and Social Functioning (respectively, *p* = 0.013, *p* = 0.016, *p* = 0.006), while results were similar for other subscales (Table 4).

**Table 4:** Distribution of Short Form-36 and Hepatitis B Quality of Life subscale scores of patients with hepatitis B by disease status at study entry

	Group-1 (*n* = 1529)	Group-2 (*n* = 1721)	Group-3 (*n* = 1007)	*p* value	All patients (*n* = 4257)
SF-36					
Physical functioning	90 (70;100)	85 (60;100)	90 (65;100)	**<0,001** ^┼, ╪^,0,203^§^	90 (65;100)
Role physical,	100 (50;100)	100 (25;100)	100 (25;100)	**<0,001** ^┼, ╪^,0,721^§^	100 (50;100)
Role emotional	100 (33,3;100)	100 (33,3;100)	100 (33,3;100)	**0,001** ^┼^,**0,004** ^╪^, 0,859^§^	100 (33,3;100)
Vitality	50 (45;70)	55 (45;70)	55 (40;70)	0,060^┼^,0,167^╪^,0,819^§^	55 (40;70)
Mental health	72 (52;84)	68 (52;80)	68 (52;80)	**0,005** ^┼^,**<0,001** ^╪^, **0,016** ^§^	68 (52;84)
Social functioning	87,5 (62,5;100)	87,5 (62,5;100)	87,5 (50;100)	**0,012** ^┼^,**<0,001** ^╪^,**0,006** ^§^	87,5 (62,5;100)
Bodily pain	100 (67,5;100)	90 (67,5;100)	90 (65;100)	0,185^┼^, **<0,001** ^╪^,**0,013** ^§^	90 (67,5;100)
General health	65 (45;75)	60 (45;70)	60 (45;75)	**<0,001** ^┼, ╪^,0,775^§^	60 (45;75)
Physical component score	79 (58,5;86)	74 (54,5;84)	74 (54;85)	**<0,001** ^┼, ╪^,0,644^§^	75,5 (55,5;85)
Mental component score	74 (52,4;81,8)	69,4 (50;80)	67,8 (49;80,8)	**<0,001** ^┼,╪^,0,511^§^	70,6 (50,3;80,8)
HBQOL					
Psychological Well-Being	90 (67,5;97,5)	85 (70;95)	82,5 (65;95)	**0,019** ^┼^,**<0,001** ^╪^,0,090^§^	85 (67,5;95)
Anticipation anxiety	80 (56,7;93,3)	76,6 (53,3;90)	70 (50;90)	**0,008** ^┼^, **<0,001** ^╪^, **0,001** ^§^	76,6 (53,3;93,3)
Vitality	80 (64;88)	76 (64;88)	76 (60;88)	**<0,001** ^┼^,**0,001** ^╪^,0,758^§^	80 (64;88)
Stigma	93,3 (73,3;100)	90 (73,3;100)	90 (70;100)	0,056^┼^, **0,001** ^╪^,0,071^§^	90 (73,3;100)
Vulnerability	80 (60;93,3)	80 (60;93,3)	73,3 (53,3;93,3)	**<0,001** ^┼,╪^,0,300^§^	80 (60;93,3)
Total score	85 (67,1;93,6)	80,7 (66,4;91,4)	79,3 (63,9;90,7)	**<0,001** ^┼,╪^,**0,024** ^§^	81,4 (65,7;92,1)

*SF-36* Short Form-36, *HBQOL* Hepatitis B Quality of Life, Median (25 %, 75 %) are presented, Bonferroni correction was used, ^┼^significant difference was obtained in comparing group 1 and 2, ^╪^significant difference was observed between group 1 and 3, ^§^significant difference was observed between group 2 and 3, bold data reflected a statistical significance

### HBQOL results

#### HBQOL results according to patient properties

The relationship of the obtained HBQOL scores with potential markers was studied. In male and married patients who did not receive CHB therapy, and without another chronic disease present and who were followed-up at a training hospital and were found to be HBeAg negative, it was observed that the total score was significantly higher when compared to other groups (*p* < 0.05 for all). Variances determined in the distribution of scores according to the properties of patients regarding their HBQOL subscales are presented in Table 5. We determined that age did not have an effect on Psychological Well-Being, or total scores, while scores in other subscales demonstrated an increase associated with age. We also observed that the period of therapy had no effect on the total score and all the subscale scores. Only the subscale scores of Stigma were increased in case of longer periods after the diagnosis of the disease, while other subscales were not affected. It was also determined that there is a reverse correlation between elevated ALT levels and all subscale scores and in the total score of the HBQOL scale. A relationship between HBV DNA level and Psychological Well-Being and total score was not found; however, a positive correlation was found between HBV DNA levels and Vitality subscale while there was a negative correlation between HBV DNA levels and other subscales (Table 3).

**Table 5:** Distribution of Hepatitis B Quality of Life subscale scores by some socio-demographic and disease-related characteristics of patients^a^

Variable	Psychological Well-Being	Anticipation anxiety	Vitality	Stigma	Vulnerability	Total score
Gender	***p*** **:<0,001**	***p*** **:<0,001**	***p*** **:<0,001**	*p*:0,921	***p*** **:0,003**	***p*** **:0,001**
Male	87,5 (70;97,5)	76,7 (56,7;93,3)	80 (64;92)	90 (73,3;100)	80 (60;93,3)	82,8 (67,1;92,8)
Female	85 (65;95)	73,3 (50;93,3)	76 (60;88)	90 (73,3; 100)	80 (60;93,3)	80 (63,6;91,4)
Marriage status	***p*** **:0,001**	***p*** **:<0,001**	*p*:0,341	***p*** **:<0,001**	***p*** **:<0,001**	***p*** **:0,002**
Married	87,5 (70;95)	76,7 (56,7;93,3)	80 (64;88)	90 (73,3;100)	80 (60;93,3)	82,1 (67,1;92,8)
Single	82,5 (65;95)	73,3 (50;90)	76 (60;92)	86,7 (66,7;100)	73,3 (53,3;93,3)	78,6 (62,8;90,7)
Education level	*p*:0,441	***p*** **:0,121**	***p*** **:<0,001**	*p*:0,001	*p*:0,089	*p*:0,881
Elementery school and under	87,5 (67,5;95)	76,7 (53,3;93,3)	76 (60;88)	93,3 (73,3;100)	80 (60;93,3)	81,4 (65,7;93,6)
Middle school	82,5 (68;95)	73,3 (50;90)	76 (64;88)	86,7 (66,7;100)	80 (53,3;93,3)	80,7 (62,8;91,4)
High school	87,5 (70;95)	76,7 (54,2;90)	80 (64;92)	90 (73,3;100)	80 (60;93,3)	82,1 (67,8;92,8)
University	85 (70;97,5)	76,7 (56,6;90)	80 (68;92)	90 (73,3;100)	80 (60;93,3)	82,1 (67,1;91,4)
Regular income	***p*** **:0,005**	***p*** **:<0,001**	***p*** **:<0,001**	***p*** **:<0,001**	***p*** **:<0,001**	*p*:0,077
Yes	90 (72,5;97,5)	80 (60;93,3)	80 (64;92)	93,3 (76,7;100)	80 (60;93,3)	84,3 (70;93,6)
No	80 (62,5;95)	70 (46,7;86,7)	72 (56; 88)	86,7 (66,7;100)	73,3 (53,3;93,3)	77,1 (60,7;89,3)
Family members with hepatitis B	*p*:0,328	***p*** **:0,015**	***p*** **:<0,001**	*p*:0,082	***p*** **:0,002**	*p*:0,097
Present	85 (67,5;95)	73,3 (53,3;90)	76 (60;88)	90 (73,3;100)	80 (60;93,3)	80,7 (65;91,4)
Not	87,5 (67,5;97,5)	76,7 (54,2;93,3)	80 (64;92)	90 (73,3;100)	80 (60;93,3)	82,9 (67,1;92,8)
Received treatment for hepatitis B	***p*** **:0,001**	*p*:0,070	*p*:0,179	*p*:0,066	*p*:0,917	***p*** **:0,011**
Yes	85 (70;95)	76,7 (53,3;90)	76 (64;88)	90 (73,3;100)	80 (60;93,3)	80,7 (66,4;91,4)
No	87,5 (67,5;95)	76,7 (53,3;93,3)	80 (64;88)	90 (73,3;100)	80 (60; 93,3)	82,1 (65;92,8)
Other chronic disease	***p*** **:0,003**	*p*:0,385	***p*** **:<0,001**	*p*:0,988	***p*** **:0,010**	***p*** **:<0,001**
Present	85 (65;95)	76,7 (53,3;93,3)	72 (56;88)	90 (73,3;100)	80 (53,3;93,3)	79,3 (63,6;91,4)
Not	87,5 (70;95)	76,7 (53,3;93,3)	80 (64;92)	90 (73,3;100)	80 (60;93,3)	82 (66,4;92,1)
Hospital type	***p*** **:<0,001**	***p*** **:<0,001**	***p*** **:<0,001**	***p*** **:<0,001**	***p*** **:<0,001**	***p*** **:<0,001**
Training hospital	90 (72,5;95)	80 (60;93,3)	84 (68;88)	93,3 (76,7;100)	80 (66,7;93,3)	85,7 (70,7;93,6)
University hospital	85 (65;97,5)	73,3 (50;86,7)	76 (60;88)	90 (70;100)	73,3 (53,3;93,3)	80 (63,6;90)
Other	80 (62,5;95)	70 (46,7;86,7)	72 (56;88)	86,7 (63,3;100)	73,3 (53,3;86,7)	76,4 (59,3;88,6)
HBeAg status	***p*** **:<0,001**	*p*:0,098	***p*** **:0,015**	***p*** **:0,022**	***p*** **:<0,001**	***p*** **:<0,001**
Positive	82,5 (65;95)	73,3 (50;90)	76 (60;88)	86,7 (66,7;100)	73,3 (53,3;93,3)	79,3 (62,9;91,4)
Negative	87,5 (67,5;95)	76,7 (53,3;93,3)	80 (64;88)	90 (73,3;100)	80 (60;93,3)	82,1 (66,4;92,1)

^a^Values were not normally distributed; thus; median (% 25; % 75) scores are presented in cells, bold data reflected a statistical significance

#### HBQOL results according to patient groups

The total score and all the subscale scores of this scale had higher levels in Group 1 patients. Excluding the Stigma subscale (*p* = 0.056) between Group 1 and Group 2 patients, a significant difference was observed for subscales and total scores. However, a significant difference was found in all subscales and total scores between Group 1 and Group 3 patients (*p* < 0.05 for all). There was a significant difference in the Anticipation Anxiety subscale (*p* < 0.001) and total scores (*p* = 0.024) between Group 2 and Group 3 patients, while scores determined in other subscales were similar (Table 4).

In multivariate analysis (Table 6), HBQOL total score was statistically significantly associated with activity of the disease, controlling for gender, marital status, presence of at least one other chronic disease, current treatment status and serum ALT level.

**Table 6:** Covariance analysis of factors associated with Hepatitis B Quality of Life total score among all study participants

Variables included in the Model	Mean square	F	Sig.
Gender	8186,916	26,001	<0,001
Marriage status	6385,542	20,280	<0,001
Other chronic disease	4789,179	15,210	<0,001
HBeAg status	480,405	1526	0,217
ALT level	2547,493	8091	0,004
Hospital type	716,785	2276	0,131
Received treatment for hepatitis B	1020,020	3240	0,072
Inactive disease	3017,300	9583	0,002

R2 = 0,048 (Adjusted R2 = 0,046), Dependent Variable: total scores, *ALT* Alanine aminotransferase

## Discussion

HRQOL of CHB patients may vary according to their socio-demographic properties. In studies conducted using different scales, it was determined that educational level [6], age [10], education period [11], annual income level [10], duration of the disease and receipt of antiviral therapy [10] affected HRQOL. In this study, HRQOL scores were found to be significantly lower in women, singles, and patients with other chronic diseases. Regarding other important non-hepatitis health issues, quality of life had been reported to be reduced in women [19–21], singles [22] and patients with another chronic disorder [19, 23]. It is well known that women, in many parts of the world, receive less social support when compared to men when they experience chronic disorders. Additionally, their access to medical care is generally delayed compared with men and they are either obliged to work or take over their responsibilities before they get completely well [24]. These variations between genders may cause quality of life to be worse in female patients with CHB. Apart from these, being married may provide social, psychological and economic support and may improve the opportunity to live healthier [25, 26]. Therefore, singles may have a difficult time overcoming long-term disease and their quality of life may rapidly deteriorate. In some cases, comorbidities may reduce HRQOL more than the primary disorder [27]. Furthermore, the frequency of a referral to a hospital increases with more comorbidity [28]. The quality of life can deteriorate in CHB patients due to an increase in physical limitations because of another disorder leading to a decrease in the ability to become self-sufficient, utilization of multiple therapies or receiving more medical care.

Based on our data, we found that the quality of life of patients might decrease due to activation of the disease when factors that have an effect on HRQOL are further analyzed. Serum ALT level usually appears to be higher in active CHB patients [29]. HRQOL had significant relationship with ALT levels in our study. Accordingly, we think that active patients could have psychiatric consequences of the disease. Because these patients are frequently called back for control visits to check the activity of the disease, this may somehow lead to an increase in anxiety among these patients.

In our study, one can easily notice the presence of body pain experienced by patients who were receiving therapy during the active term of the disease. On the other hand, this group also had a better status of mental and social functionality and a decrease in anxiety. Additionally, the total scores obtained from the HBQOL questionnaire have demonstrated that patients with active disease undergoing therapy had increased quality of life. The main purpose and scope of current therapies is to avoid the formation of complications due to CHB [29]. However, these results reveal that antiviral therapies provided confidence to patients and increased their quality of life. This result can be assumed as an additional success of antiviral therapy.

According to the results we obtained from the SF-36 scale, not all of the CHB patients enrolled in our study felt themselves to be energetic. This result may also indicate evidence of subjective symptoms of patients, such as “weakness, getting tired quickly and desire to sleep for a long time”. However, when patients are assessed in accordance with the results obtained from the HBQOL questionnaire, patients feel well via spiritual means and have an intense desire to live. Nevertheless, even though patients were considered to be in a positive mental condition, they were somehow anxious about matters in the future, and furthermore the majority of patients did not have stigmata or vulnerability emotions. When these data were assessed together, we determined that the anxious status of CHB patients was not related to the community, but highly focused on medical issues. In the present study, we can admit that the HRQOL of patients that participated in the study related to CHB was considerably better when a total median score of >80 were considered.

Our study is one of the largest studies conducted in Turkey where HRQOL scores have been studied in CHB patients who are carefully monitored, and can be considered valuable due to the data obtained from patients who resided in different geographical regions and who were monitored in different types of hospitals. Even though its validity was previously tested in a Turkish population, there have been no studies published where a HBQOL scale was utilized.

Showing attention to some limitations of our study regarding the planning process could be helpful for future studies. Nevertheless, the results obtained from this study may not be generalizable to all CHB patients in Turkey. As the HBQOL was not developed for the population of cirrhotic patients, this group of patients was not included in the study. As the subscale of transmissibility is not appropriate to be used in the Turkish population, we do not recommend using the current HBQOL scale developed as is. Another limitation encountered in our study was that it may be impossible to determine the status effect ability related to the type of therapy since the HRQOL was not planned to be used as an assessment according to the type of therapy. The cross-sectional qualification and design of the current study, progression of the disease in patients and variance in the quality of life among patients during the monitoring period has limited the collection of data.

## Conclusions

The quality of life of CHB patients in the study group was found to be relatively high when assessed with the values obtained by the SF-36 and HBQOL scales. The quality of life is dependent on factors related to a number of individual factors and the course of the disease.

The quality of life can become negatively affected once the activity of the disease is increased. However, appropriate antiviral therapy can provide a higher quality of life in this group of patients. Proper assessment and management of CHB along with psychiatric support for female or single patients with active disease and comorbidities should be taken into consideration.

## Abbreviations

ALT: Alanine aminotransferase
CHB: Chronic hepatitis B
DNA: Deoxyribonucleic acid
HBQOL: Hepatitis B Quality of Life
HBV: Hepatitis B virus
HCV: Hepatitis C virus
HRQOL: Health-related quality of life
SF-36: Short Form-36
SQ: Standardized questionnaire
